# Emergence of a novel subclade of influenza A(H3N2) virus in London, December 2016 to January 2017

**DOI:** 10.2807/1560-7917.ES.2017.22.8.30466

**Published:** 2017-02-23

**Authors:** Heli Harvala, Dan Frampton, Paul Grant, Jade Raffle, Ruth Bridget Ferns, Zisis Kozlakidis, Paul Kellam, Deenan Pillay, Andrew Hayward, Eleni Nastouli, For the ICONIC Consortium

**Affiliations:** 1 Department of Clinical Virology, University College London Hospitals NHS Foundation Trust, London, United Kingdom; 2 Department of Infection and Immunity, University College of London, London, United Kingdom; 3 NIHR UCLH/UCL Biomedical Research Centre, London, United Kingdom; 4 Department of Medicine, Imperial College Faculty of Medicine, London, United Kingdom; 5 Department of Infectious Disease Informatics, Farr Institute of Health Informatics Research, London, United Kingdom; 6 Department of Population, Policy and Practice, UCL GOS Institute of Child Health, London, United Kingdom; 7 The members of these networks are listed at the end of the article

**Keywords:** influenza, outbreak, next generation sequencing, molecular

## Abstract

We report the molecular investigations of a large influenza A(H3N2) outbreak, in a season characterised by sharp increase in influenza admissions since December 2016. Analysis of haemagglutinin (HA) sequences demonstrated co-circulation of multiple clades (3C.3a, 3C.2a and 3C.2a1). Most variants fell into a novel subclade (proposed as 3C.2a2); they possessed four unique amino acid substitutions in the HA protein and loss of a potential glycosylation site. These changes potentially modify the H3N2 strain antigenicity.

The ongoing influenza season started early in eleven European Union countries, including England, on week 46 of 2016 [1]. The majority of reported infections have been caused by clade 3C.2a or 3C.2a1 influenza A(H3N2) viruses. The clade 3C.2a contains the current vaccine strain A/Hong Kong/4801/2014, and the first few viruses within the more recently emerged subclade 3C.2a1 were earlier shown to be antigenically matched with the vaccine component [2]. However, evidence for suboptimal vaccine effectiveness (VE) against laboratory-confirmed influenza A infection in people over 65 years-old was obtained in the first studies from Finland [3] and Sweden [4].

An outbreak of influenza A(H3N2) was first notified in our London centre on 30 December 2016. The outbreak coincided with unusually high ongoing circulation of respiratory syncytial virus (RSV) (Figure 1), and affected both patients and staff in the acute medical unit (AMU).

**Figure 1:**
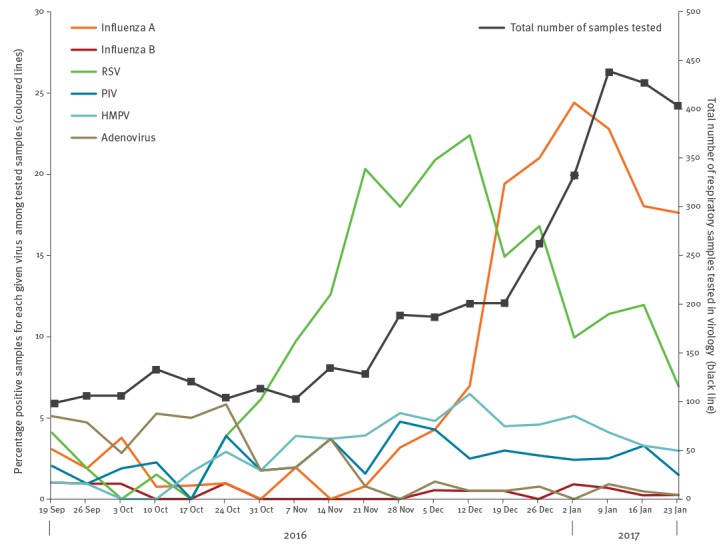
Percentage of positive respiratory samples for given viruses, and total number of respiratory samples tested per week, at the Department of Clinical Virology, University College of London Hospital, 19 September 2016–30 January 2017 (n=1,690 samples)

Adeno: adenovirus; HMPV: human metapneumovirus; PIV: parainfluenza virus; RSV: respiratory syncytial virus.

While infection control precautions were intensified, it resulted in multiple bay closures. We suspected that the sharp increase in the number of influenza A(H3N2) infections may have been caused by the emergence of a new genetic variant of H3N2, a hypothesis investigated through next generation sequencing (NGS) of influenza A(H3N2) strains.

## Collection and analysis of respiratory samples

The main study was based on respiratory samples (n = 1,690) analysed at the Department of Virology, University College of London Hospital (UCLH), United Kingdom between 21 December 2016 and 24 January 2017. Most samples were collected as part of routine diagnostics from inpatients and patients seen at the Accident and Emergency department, and to a lesser extent from outpatients. The basic epidemiological data including patients’ age, admission and sampling dates as well as data on intensive care unit (ICU) admissions and deaths were collected. For comparison, results from influenza A and other virus testing from UCLH since 19 September 2016 were also analysed. The study was approved by the NRES Committee London, Surrey Borders HRA, (REC reference: 13/LO/1303).

All samples were initially screened for influenza A virus by reverse transcription-PCR targeting the matrix gene. A total of 67 influenza A(H3N2) virus-positive samples obtained between 8 December 2016 and 3 January 2017 were sequenced. RNA was amplified using a modified eight-segment method [5]. Library preparations were generated as previously described [5,6]. A neighbour joining phylogenetic tree was constructed using Molecular Evolutionary Genetics Analysis (MEGA) 6 software [7]. Some sequences in the phylogenetic analysis were from the Global Initiative on Sharing All Influenza Data (GISAID); the authors gratefully acknowledge the 36 originating and submitting laboratories who contributed sequences to GISAID (www.gisaid.org). 

## Characteristics of the influenza A(H3N2) outbreak

**Figure 2:**
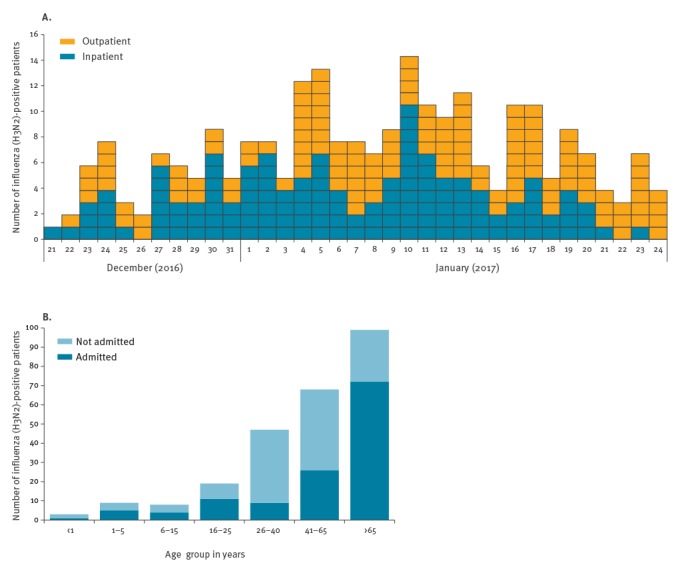
Number (A) and age distribution (B) of influenza A(H3N2)-positive patients diagnosed at the University College of London Hospitals, 16 December 2016–24 January 2017 (n=253 patients)

Of the 1,690 respiratory samples obtained between 21 December 2016 and 24 January 2017, 352 samples were positive for influenza A(H3N2) virus (21%; Figure 1). Of those, 294 influenza A(H3N2)-positive samples had been obtained from 253 UCLH patients. Of patients with influenza A(H3N2) infection, over 50% (128/253) required hospital admission. An average of three inpatients (either existing inpatients or new admissions) were identified as influenza A(H3N2)-positive each day, and the highest number of hospital admissions was recorded on 10 January (n = 11; Figure 2a). Over the outbreak period, six patients required ICU admission and five died. Over a third of influenza A(H3N2) infections were seen in adults over 65 years-old (99/253; 39%), most of them admitted to hospital (72/99; 73%, Figure 2b).

## Description of influenza A(H3N2) viruses circulating in London

**Figure 3:**
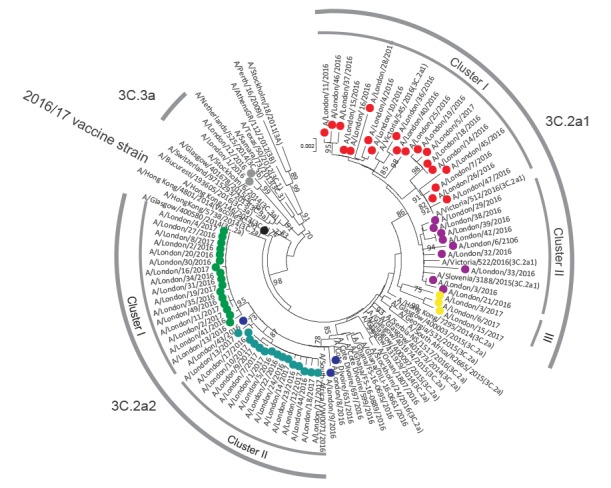
Phylogenetic tree of the haemagglutinin gene sequences of virus strains recovered in this study using reference viruses for the different phylogenetic influenza A(H3N2)clades (n = 103 sequences)

Phylogenetic analyses of haemagglutinin (HA) sequences indicated co-circulation of variants from subclades of 3C.3a (n = 2), 3C.2a1 (n = 31) and 3C.2a (n = 34) (Figure 3).

Bootstrap values obtained with 100 replicates are shown on some branches of the neighbour joining tree. The scale represents the percentage of nucleotide substitutions. Sequences are annotated in the following way: influenza type/geographical area of sample collection/sample number/year. For sequences previously characterised as belonging to the subclade 3C.2a, this is indicated after the year in parentheses. London sequences retrieved in this study are indicated with coloured circles (n = 67), and the vaccine strain with a black circle.

The authors gratefully acknowledge the 36 originating and submitting laboratories who contributed sequences used in the phylogenetic analysis to the Global Initiative on Sharing All Influenza Data (GISAID; www.gisaid.org).

Interestingly, our 3C.2a virus strains differed from the previously characterised subclade 3C.2a strains as well as from subclade 3C.2a1, and hence we have proposed them as a new subclade 3C.2a2. This subclade in turn split into two well defined but internally homogenous sub-clusters (cluster I and II; Figure 3), and also included all suspected outbreak cases admitted to AMU between 27 December 2016 and 3 January 2017 (n = 15).

**Figure 4:**
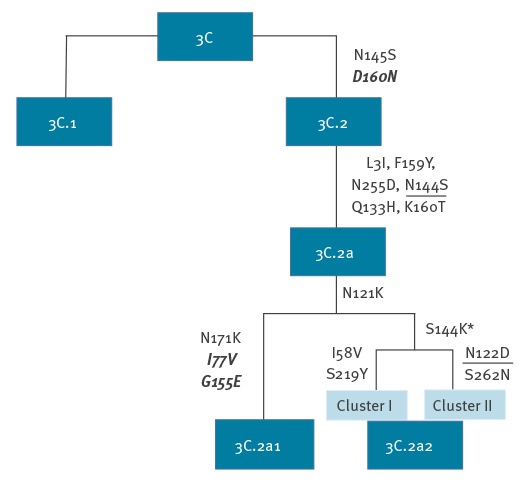
Schematic diagram demonstrating the shared haemagglutinin (HA) amino acid changes between clades 3c, 3C.2, 3C.2a, 3C.2a1 and 3C.2a2 based on HA1 and HA2 numbering

Individual clades of influenza A are typically defined by amino acid substitutions that occur as they diversify from parental strains. Such substitutions are potentially functionally relevant as they may influence the antigenicity and susceptibility to neutralising antibody induced by infection with other lineages of H3N2. Thus, investigated variants within the subclades 3C.2a and 3C.2a1 (n = 65) inherited amino acid substitutions known to define their parental clades (Figure 4). All variants within the proposed subclade 3C.2a2 shared two substitutions N121K and S144K, whereby S144K is an antigenic site flanking the receptor binding site (RBS). A further two additional substitutions were observed in each 3C.2a2 cluster (I58V and S219Y in cluster I and N122D and S262N in cluster II), all in the HA1 region and based on HA1 numbering. Cluster II viruses lost the potential N-linked glycosylation site (N122D).

Changes in HA1 are indicated in normal font while changes in HA2 are in bold and italic. Changes in antigenic sites are shown with an asterisk (*) while changes resulting in the loss of a potential N-linked glycosylation site are underlined.

## Discussion

In our centre in London, the early start and higher intensity of the 2016/17 influenza A(H3N2) virus epidemic mirrored that of the season 2014/15 where the subtype H3N2 also predominated. During the 2014/15 season, most influenza A(H3N2) infections in Europe were shown to be caused by antigenically drifted virus variants within the new genetic subgroup 3C.2a [8]. Our genetic analysis of London A(H3N2) viruses demonstrates ongoing co-circulation of drifted variants from multiple subclades (3C.3a, 3C.2a1 and proposed 3C.2a2). Four or more substitutions in two or more antibody binding sites are predicted to give an antigenically different virus [9] as in our case. Although we did not observe mutations in the seven positions suggested as being responsible for major transition clusters [10], position 144 is at the flank of the RBS, and additionally recognised as antigenic [11].

Although not necessarily determining major antigenic drift, the alterations of N-linked glycosylation sites are likely to contribute to more complex conformational changes in the HA due to gain or loss of glycosylation and can thus facilitate immune escape [12]. Furthermore, any amino acid changes in the 140–146 region of HA have been shown to be characteristic for antigenically distinct viruses of epidemic significance [9,13,14]. The amino acid substitution S144K in the emerging subclade 3C.2a2 viruses together with the loss of an N-linked glycosylation site (N122D) shows potential for antigenic drift that warrants further monitoring during this ongoing season. A limitation of our study was the lack of detailed vaccination data.

Our findings in London of the rapid emergence of genetically drifted influenza A(H3N2) viruses underscore the potential for such strains to spread rapidly in hospital environments among patients and staff. Characterising emerging strains of influenza by next generation sequencing adds to the local and national monitoring of influenza trends. Further studies are needed to investigate the antigenic effects of substitutions occurring within the newly described subclade.

## Notes

**The ICONIC Consortium:** Tiziano Gallo Cassarino, Myrto Kremyda-Vlachou, Ruth Blackburn, Catherine Smith, Duncan Clark, Steven Morris, Andrew Leigh-Brown, Anne Johnson.
