# Addendum for Euro Surveill. 2017;22(8)

**DOI:** 10.2807/1560-7917.ES.2017.22.40.171005

**Published:** 2017-10-05

**Authors:** Eurosurveillance editorial team

**Affiliations:** 1 European Centre for Disease Prevention and Control, Stockholm, Sweden

To the article entitled ‘Emergence of a novel subclade of influenza A(H3N2) virus in London, December 2016 to January 2017’ by H Harvala et al., published on 23 February 2017, the following information is added at the request of the authors.

We gratefully acknowledge the authors, originating and submitting laboratories of the sequences from GISAID’s EpiFlu Database on which this research is based. The list is detailed in the Table below.

**Table:** Information on GISAID sequences on which the study is based

Virus isolate	Segment ID/ Accession number	Country	Collection date (year-month-day)	Originating laboratory	Submitting laboratory
A/Stockholm/18/2011	EPI326139	Sweden	2011-03-28	Public Health Agency of Sweden, Solna, Sweden	National Institute for Medical Research, London, UK
A/Perth/16/2009	EPI182941	Australia	2009-04-07	Pathwest QE II Medical Centre, Nedlands, Australia	WHO Collaborating Centre for Reference and Research on Influenza, Victoria, Australia
A/AthensGR/112/2012	EPI358885	Greece	2012-02-01	Hellenic Pasteur Institute, Athens, Greece	National Institute for Medical Research, London, UK
A/Texas/50/2012	EPI391247	United States	2012-04-15	Texas Department of State Health Services, Austin, USA	Centers for Disease Control and Prevention, Atlanta, US
A/Samara/73/2013	EPI460558	Russian Federation	2013-03-12	WHO National Influenza Centre, Saint Petersburg, Russian Federation	National Institute for Medical Research, London, UK
A/Netherlands/525/2014	EPI574644	Netherlands	2014-12-17	National Institute for Public Health and the Environment (RIVM), Bilthoven, Netherlands	National Institute for Medical Research, London, UK
A/Stockholm/6/2014	EPI530677	Sweden	2014-02-06	Public Health Agency of Sweden, Solna, Sweden	National Institute for Medical Research, London, UK
A/Glasgow/401678/2015	EPI574607	United Kingdom	2015-01-07	Gart Naval General Hospital, Glasgow, United Kingdom	National Institute for Medical Research, London, UK
A/Switzerland/9715293/2013	EPI530687	Switzerland	2013-12-06	Hopital Cantonal Universitaire de Geneves, Geneve, Switzerland	National Institute for Medical Research, London, UK
A/Bucuresti/193605/2016	EPI769485	Romania	2016-03-08	Cantacuzino Institute, Bucharest, Romania	Crick Worldwide Influenza Centre, London, UK
A/Hong Kong/146/2013	EPI426061	Hong Kong (SAR)	2013-01-11	Government Virus Unit, Kowloon, Hong Kong (SAR)	National Institute for Medical Research, London, UK
A/Hong Kong/4801/2014	EPI834581	Hong Kong (SAR)	2014-02-26	National Institute for Medical Research, London, UK	Centers for Disease Control and Prevention, Atlanta, US
A/Hong Kong/5738/2014	EPI539806	Hong Kong (SAR)	2014-04-30	Government Virus Unit, Kowloon, Hong Kong (SAR)	National Institute for Medical Research, London, UK
A/Glasgow/400580/2014	EPI574601	United Kingdom	2014-12-26	Gart Naval General Hospital, Glasgow, United Kingdom	National Institute for Medical Research, London, UK
A/South Africa/vw0071/2016	EPI829363	South Africa	2016-07-21	National Institute for Communicable Diseases, Sandringham, South Africa	Crick Worldwide Influenza Centre, London, UK
A/Cote DIvoire/651/2016	EPI781608	Cote d'Ivoire	2016-04-25	Pasteur Institut of Côte d'Ivoire, Abidjan, Cote d'Ivoire	Crick Worldwide Influenza Centre, London, UK
A/Cote DIvoire/697/2016	EPI781616	Cote d'Ivoire	2016-05-04	Pasteur Institut of Côte d'Ivoire Abidjan, Cote d'Ivoire	Crick Worldwide Influenza Centre, London, UK
A/Cote DIvoire/599/2016	EPI781602	Cote d'Ivoire	2016-04-15	Pasteur Institut of Côte d'Ivoire Abidjan, Cote d'Ivoire	Crick Worldwide Influenza Centre, London, UK
A/Ghana/FS-16-0889/2016	EPI829309	Ghana	2016-06-26	Noguchi Memorial Institute for Medical Research, Accra, Ghana	Crick Worldwide Influenza Centre, London, UK
A/Ghana/FS-16-0695/2016	EPI829307	Ghana	2016-05-23	Noguchi Memorial Institute for Medical Research, Accra, Ghana	Crick Worldwide Influenza Centre, London, UK
A/Ghana/DILI-16-0661/2016	EPI829305	Ghana	2016-06-27	Noguchi Memorial Institute for Medical Research, Accra, Ghana	Crick Worldwide Influenza Centre, London, UK
A/Stockholm/24/2016	EPI781630	Sweden	2016-02-22	Public Health Agency of Sweden, Solna, Sweden	Crick Worldwide Influenza Centre, London, UK
A/Oman/1807/2016	EPI769519	Oman	2016-02-12	Central Public Health Laboratory, Muscat, Oman	Crick Worldwide Influenza Centre, London, UK
A/Glasgow/431929/2014	EPI574704	United Kingdom	2014-11-23	Gart Naval General Hospital, Glasgow, United Kingdom	National Institute for Medical Research, London, UK
A/Glasgow/400097/2015	EPI574599	United Kingdom	2015-01-02	Gart Naval General Hospital, Glasgow, United Kingdom	National Institute for Medical Research, London, UK
A/Glasgow/401674/2015	EPI574605	United Kingdom	2015-01-01	Gart Naval General Hospital, Glasgow, United Kingdom	National Institute for Medical Research, London, UK
A/Glasgow/401673/2014	EPI574603	United Kingdom	2014-12-29	Gart Naval General Hospital, Glasgow, United Kingdom	National Institute for Medical Research, London, UK
A/Serbia-NS/1517/2016	EPI746057	Serbia	2016-02-10	I Institute of Public Health, Novi Sad, Serbia	Crick Worldwide Influenza Centre, London, UK
A/South Africa/R2665/2015	EPI630781	South Africa	2015-05-25	National Institute for Communicable Diseases, Sandringham, South Africa	Crick Worldwide Influenza Centre, London, UK
A/Georgia/532/2015	EPI589699	Georgia	2015-03-09	National Centre for Disease Control and Public Health, Tbilisi, Georgia	Crick Worldwide Influenza Centre, London, UK
A/Glasgow/400003/2015	EPI574597	United Kingdom	2015-01-02	Gart Naval General Hospital, Glasgow, United Kingdom	National Institute for Medical Research, London, UK
A/Hong Kong/7295/2014	EPI551882	Hong Kong (SAR)	2014-08-07	Government Virus Unit, Kowloon, Hong Kong (SAR)	National Institute for Medical Research, London, UK
A/Slovenia/3188/2015	EPI699750	Slovenia	2015-12-26	Laboratory for Virology, National Institute of Public Health, Ljubljana, Slovenia	Crick Worldwide Influenza Centre, London, UK
A/Victoria/522/2016	EPI831759	Australia	2016-07-04	Monash Medical Centre, Victoria, Australia	WHO Collaborating Centre for Reference and Research on Influenza, Victoria, Australia
A/Victoria/512/2016	EPI831633	Australia	2016-07-13	Monash Medical Centre, Victoria, Australia	WHO Collaborating Centre for Reference and Research on Influenza, Victoria, Australia
A/Victoria/545/2016	EPI831876	Australia	2016-07-31	Monash Medical Centre, Victoria, Australia	WHO Collaborating Centre for Reference and Research on Influenza, Victoria, Australia

GISAID: Global Initiative on Sharing All Influenza Data; SAR: Special Administrative Regions of the People's Republic of China; UK: United Kingdom; US: United States; WHO: World Health Organization.

All submitters of data may be contacted directly via the GISAID website www.gisaid.org.

